# Sequential visual stimuli increase high frequency power in the visual cortex

**DOI:** 10.1038/s41598-026-52253-9

**Published:** 2026-05-17

**Authors:** Julian Keil, Victor Hernandez-Urbina, Chrystalleni Vassiliou, Camin Dean, Dietmar Schmitz, Jens Kremkow, Jérémie Sibille

**Affiliations:** 1 Department of Cognitive Science, University of Potsdam, 14469 Potsdam, Germany; 2 Nuuron GmbH Berlin, Berlin, Germany; 3 Charité-Universitätsmedizin Berlin, Corporate Member of Freie Universität Berlin and Humboldt-Universität Berlin, Clinical Neurotechnology Laboratory, Department of Psychiatry and Neurosciences, 10117 Berlin, Germany; 4 German Center for Neurodegenerative Diseases (DZNE) Berlin, 10117 Berlin, Germany; 5 Charité-Universitätsmedizin Berlin, Corporate Member of Freie Universität Berlin and Humboldt-Universität Berlin, Neuroscience Research Center, 10117 Berlin, Germany; 6 Charité-Universitätsmedizin Berlin, Corporate Member of Freie Universität Berlin and Humboldt-Universität Berlin, Einstein Center for Neurosciences, 10117 Berlin, Germany; 7 Charité-Universitätsmedizin Berlin, Corporate Member of Freie Universität Berlin and Humboldt-Universität Berlin, Bernstein Center for Computational Neuroscience, 10115 Berlin, Germany; 8 Charité-Universitätsmedizin Berlin, Corporate Member of Freie Universität Berlin and Humboldt-Universität Berlin, NeuroCure Cluster of Excellence, 10117 Berlin, Germany; 9 Charité-Universitätsmedizin Berlin, Corporate Member of Freie Universität Berlin and Humboldt-Universität zu Berlin, Institute of Cell and Neurobiology, 10115 Berlin, Germany; 10 Institute of Biology, Otto von Guericke University Magdeburg, 39120 Magdeburg, Germany

**Keywords:** Biological techniques, Neuroscience

## Abstract

Today, flickering full-field visual stimulation is used to increase neuronal oscillations for a variety of research or therapeutic purposes. We propose spatially organized sequential visual flickering stimulation as a newer tool to circumvent the intrinsic low-pass filter nature of the vertebrate visual system, in order to increase the power of high frequency oscillations in the visual system. We show that spatially organized visual flickering can increase power in high frequencies (100 to 190 Hz) in the visual cortex of mice. Consequently, spatially organized sequential sensory stimulation should be regarded as a putative new way leading to power increases in high frequency domains.

**Supplementary Information:**

The online version contains supplementary material available at 10.1038/s41598-026-52253-9.

## Introduction

Repetitive visual stimulation can lead to synchronized firing of neurons in the visual cortex in the stimulation frequency, so-called steady-state visual evoked potentials (SSVEP)^1^. This method is routinely used as an experimental tool in electrophysiological research and clinical settings for low to mid frequency ranges^2^. For example, visual neuronal entrainment with 40 Hz full-field stimulation has been used as a neuroprotective approach to counteract neurodegeneration associated with Alzheimer’s disease^3,4^, slow down the disease progression^5^, and improve sleep^6^. However, the actual benefit of visual full-field 40 Hz gamma entrainment against neurodegeneration has been recently questioned by two independent studies that report a progressive attenuation of the desired 40 Hz neuronal entrainment along the visual pathway, leading to an absence of 40 Hz entrainment in the hippocampus, probably in part due to biophysical neuronal properties^7,8^. Overall, high frequency oscillation power tends to dissipate in neural tissue, which is thought to act as a low pass filter with a 1/frequency characteristic^9^. Thus, the few instances where the local field potential (LFP) power is transiently increased are of particular interest.

**Fig. 1:**
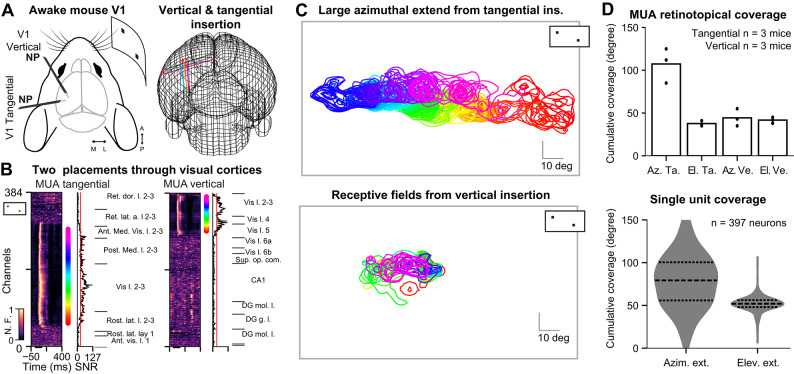
Tangential insertions capture extended azimuthal retinotopy within the visual cortex. **(A)** Schematic of a mouse passively viewing stimulations presented on a screen, with tangential and vertical Neuropixels (NP) insertions (left), and the 3D reconstruction of the brain showing probe placements (right, *n* = 6 mice). **(B)** Visually driven multi-unit-activity (MUA) evoked by black squares on white background for tangential (left) and vertical (right) probe insertions. If a channel’s signal to noise ratio (SNR), is above threshold (15 a.u.), a color dot is placed in the aligned plot right to MUA plots; its corresponding receptive field (RF) will be represented in panel C with the identical color-code. The reconstructed layer positioning of each channel is represented aligned on the right. **(C)** Receptive field coverage from tangential (top) and vertical (bottom) insertions with the inset showing the sparse noise visual stimuli used to measure these receptive fields. Note that the retinotopic coverage obtained with tangential insertions is capturing almost the entire extent of the screen which corresponds to a visual capture of more than 100° of retinotopy in a single eye within the cortex. **(D)** Quantification of the retinotopic coverage observed in the MUA (top) and in the corresponding paired receptive field extent (6 mice, 397 neurons).

**Fig. 2:**
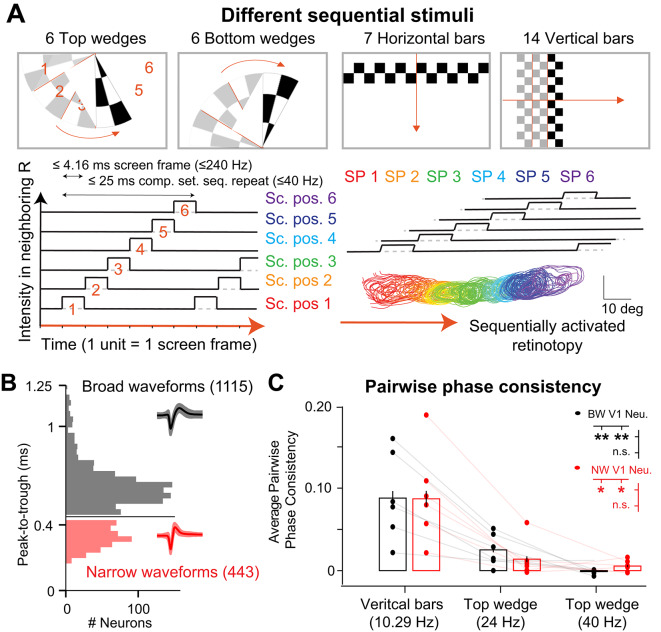
Single neuron activity is higher in response to lower sequential repeat frequencies. (**A**). Four different sequential stimuli were used: 6 top wedges, 6 bottom wedges, 7 horizontal bars, and 14 vertical bars (top). Wedge exposure is illustrated in time (bottom left) showing the corresponding sequential screen positions being stimulated overlayed with multi-unit activity within receptive fields (bottom right). Note that each sequential stimulus is exposed for one single screen frame, while responses to specific repeat frequencies are quantified using the complete set of sequential stimuli, i.e. every 6 screen frames for wedges, and every 7 or 14 screen frames for horizontal and vertical bars respectively. (**B**). Single neuronal waveforms are separated between broad (black) and narrow (red) waveforms based on their peak-to-trough durations. (**C**). Estimate of pairwise phase consistency (PPC) of broad and narrow waveform neurons in *n* = 6 different mice, for 3 different repeat frequencies (*n* = 1115 BW neurons, *n* = 443 NW neurons, pooled within each of the *n* = 6 mice, two ways repeated measures ANOVA, p = 5.1 10^− 3^ for NW, p = 2.0 10^− 3^ for BW, followed by post-hoc pairwise comparisons with Bonferroni corrections. The graph shows means and standard errors of the mean (SEM) calculated from the illustrated dots which represent average neuronal responses per mouse.

Aiming to directly enhance higher frequency activity in the brain, we propose to spatially combine neighboring flickering checkerboard patterns with very small delays along different retinotopic positions^10^(Fig. 1C and 2A). Previous research has shown that multiple frequencies can be processed in parallel to simultaneously evoke frequency-specific SSVEP in separate visual cortical areas^11^. Therefore, it should be feasible to evoke SSVEP in circumscribed cortical areas by selectively stimulating neighboring receptive fields in parallel along the retinotopic pathways. Phase-shifting the stimulation of different receptive fields would then allow evoking parallel SSVEP at the same frequency but with very short time delays between neighboring retinotopic areas. We hypothesize then that these parallel SSVEP, triggered with different phases, will be integrated laterally between processing pathways in the primary visual cortex. This would allow for the summation or non-linear integration of low-frequency SSVEP optimized to the response properties of the visual system toward enhancement of high-frequency power. Consequently, such stimulation with spatially organized sequential visual flickering would allow bypassing the low-pass filter properties of neuronal tissue. In short, these sequential visual flickering stimulations take advantage of the independence of parallel retinotopic pathways in order to activate different retinal and cortical positions with very short delays aiming to stimulate the cortex at high frequency in a predetermined way. To test this hypothesis, we presented mice with different forms of spatially organized sequential visual flickering light stimulation -tailored to their visual acuity- to examine the high-frequency power spectrum of visual cortex.

## Results

To address whether sequential stimulation can enhance high frequency power in the visual cortex, we monitored neuronal activity in the primary visual area (V1) of awake head-fixed mice using Neuropixels probes^12^ (NP). We accustomed the animals to peacefully sit and watch different visual stimulations. NP inserted in two different angles (Fig. 1A) precisely monitored visual evoked neuronal activity throughout the cortex (Fig. 1B). As routinely done in laboratories studying vision, and as detailed in previous publications^13,14^, great care was taken to well align the screen position to the measured retinotopy of the current insertion, in order to maximize retinotopic coverage (Fig. 1C). Consequently, both at the level of the Multi-Unit-Activity (Fig. 1B) and at the level of the corresponding single unit activity (Ext. Data Figs. 2 and 4) we observed visual responses over areas up to 100° extent (Fig. 1D) as classically done in the laboratory^13–17^. Four types of spatially organized sequential visual stimulations were presented in a predetermined order, at three different screen refresh frequencies (144/180/240 Hz) for 2 s long trials, in 50 consecutive trials per stimulus type and screen refresh frequency (Fig. 2A). Consequently, a given stimulus having N sequentially presented sections had a cumulative duration of N*(1000/ screen refresh frequency) ms before the first section is repeated (Fig. 2). Given the three different screen frequencies and the 3 different sequential section numbers (6, 7, 14) there were 9 different “repeat frequencies” ranging from 10.3 to 40 Hz, distributed among 12 different sequential stimulations (Fig. 2 & Ext. Data Fig. 5). In other words, repeat frequency refers to the frequency at which each individual section of the stimulus is repeated at a given screen position (Fig. 2A). For example, in a stimulus with 14 sections, at a 144 Hz screen refresh frequency, each section is repeatedly exposed at a frequency of 10.3 Hz, where neighboring sections are exposed with a phase shift of 6.94 ms (Fig. 2A, for vertical bars, 14*6.94 ms = 97.16 ms ~ 10.28 Hz). This approach is used here to explore the possibility of inducing higher frequency activity throughout the brain, starting with the visual cortex. To examine basic response properties, we first measured evoked responses to the different repeat frequencies of the sequential stimuli.

**Fig. 3:**
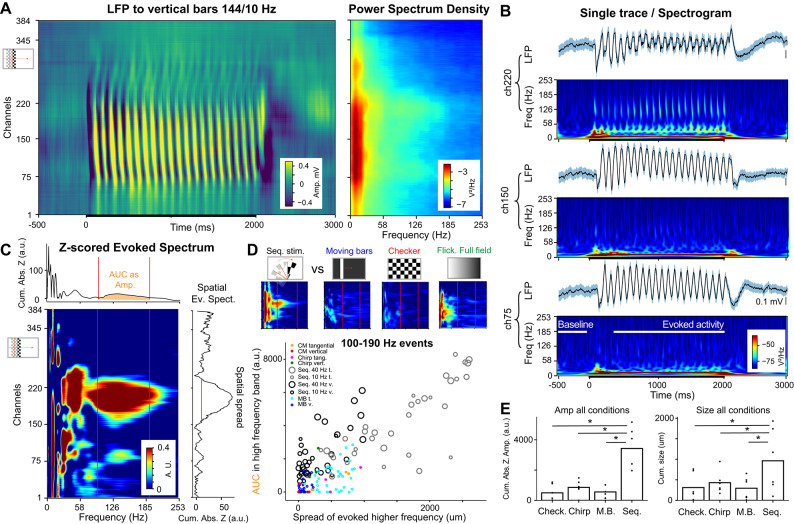
Sequential stimulations enhance high frequency (100 to 190 Hz) power in V1. **(A)** Stereotypical evoked LFP averaged across 50 trials of the 2 s sequential exposure of vertical bars (left), and the corresponding power spectral density (PSD; right), for the 144 Hz screen refresh frequency, corresponding to the 10.3 Hz repeat frequency. **(B)** Visually evoked LFP (top) and corresponding spectrum (heatmap, bottom), for three different channels along the probe; with a shaded blue area accounting for the inter-trial SEM of the obtained LFP. **(C)** Z-scored evoked spectrum based on stable evoked activity, with the corresponding cumulative absolute values in both top and right insets. These are used to quantify the strength of the evoked high frequency events by the area under the curve (AUC) and the spatial spread of the evoked high frequency events (for all values above 15 a.u. within visually driven areas). **(D)** All sequential stimuli and certain moving bar orientations enhanced higher frequency power in V1 over a large area (n = 6 mice, 3 vertical and 3 tangential insertions). The repeat frequency of each sequential stimulus, ranging from 10 to 40 Hz, is reported by the size of the point. **(E)** Averaged quantifications of the cumulative absolute Z-score value for each of the conditions: checkers, chirp, moving bar (M.B.; averaged over all 12 orientations), and sequential (Seq.; averaged over all 12 sequential stimuli,) from 6 mice, in amplitude: sequential versus resp. checker/chirp/moving bars, *p* = 0.03125/0.03125/0.03125; in size, *p* = 0.03125/0.0625/0.03125, Wilcoxon signed-rank test).

To do so, we extracted the single-unit activity from the different recordings and sorted them based on their waveform shape into either narrow waveform (NW) putative fast-spiking inhibitory neurons or broad waveform (BW) putative regular spiking excitatory neurons (Fig. 2B & Ext. Data Fig. 1). All recorded neurons were confirmed to be within visual cortices by 3D reconstruction and channel to brain region associations (Ext. Data Fig. 2). In line with previous publications^8^, we observed significant changes in single-unit firing across repeat frequencies within recordings (Ext. Data Fig. 3) In addition, we observed a significant decrease in pairwise phase consistency (PPC) for different visual stimulus types (Fig. 2D & Ext. Data Fig. 5). In particular V1 neurons show a significant PPC decrease with increasing repeat frequencies of our sequential stimulations (Fig. 2D, for repeat frequencies of 10.3/24/40 Hz, BW mean PPC = 0.0879 ± 0.0085/ 0.0251 ± 0.0030/ 0.0012 ± 0.00045 and NW mean PPC = 0.0870 ± 0.0095/ 0.0138 ± 0.00339/ 0.0055 ± 0.0011; two ways ANOVA followed by post-hoc repeated measure with Bonferroni correction p-values = 0.008/0.0023/0.162 for paired comparison between BW and 0.0149/0.018/0.24 between NW). The details for the twelve different stimulation conditions are reported in the supplementary materials (Ext. Data Fig. 5). We hypothesized that the spatially complex sequential structure of our stimulus would evoke in the cortex an equivalent activity throughout the different receptive fields, thereby inducing an artificial synthetic neuronal wavefront similarly to the known visually-evoked and spontaneous traveling waves (Fig. 2A).

To test whether sequential visual stimulation can enhance high frequency power, we analyzed the LFP evoked by the different stimulations (Fig. 3A). First, we confirmed the consistent presence of our stimulus repeat frequency in all conditions (Fig. 3A & C for vertical bars, repeat frequency = 10.3 Hz, Ext. Data Fig. 6D-E-F bottom wedges, repeat frequency = 24 Hz). Then, we quantified in each recording the evoked power spectrum in each channel (Fig. 3B), in order to extract the Z-score of the evoked spectrum (Fig. 3C) for the different sequential stimulations (along with other standard visual stimulations, Fig. 3D, Ext. Data Fig. 6). Most of the sequential stimulations enhanced high frequency power in V1 in defined locations along the recorded retinotopy (Fig. 3C-D). These enhanced high frequency activities (between 100 and 190 Hz) were often present over larger areas in our tangential recordings compared to vertical recordings (Fig. 3E). These large areas are inferred from the number of channels exhibiting high frequency power increase (approximated from ch. * 10 μm > 500 μm), mostly present during sequential stimuli in most of the animals (Ext. Data Fig. 6J, with averages of 962 ±250 μm for sequential stimuli, when having 311 ± 93 μm, 430 ± 75, and 491 ± 120 μm for checker, chirp, moving bars, *p* = 0.03125/0.0625/0.03125 respectively, Wilcoxon signed rank test, Fig. 3E); what becomes more evident when comparing tangential insertions to vertical ones (Ext. Data Fig. 6J). Their presence confirmed that spatial sequential visual stimulation produced spatially determined entrainment in the cortex. Interestingly, it seemed that lower repeat frequencies in our sequential stimulations had a better capacity to enhance such high frequency activity (Fig. 3D) which is likely the consequence of the longer exposure time e.g. 4.16 ms for 240 Hz screen refresh frequency vs. 6.94 ms at 144 Hz screen refresh frequency (Fig. 2). This effect is associated with a higher PPC at lower repeat frequencies (Fig. 2D). In order to verify that this high frequency power increase was specific to sequential stimulations, we compared the evoked high frequency activity in the very same recordings to two other classical visual stimulations: a checker stimulus, an alternating full-field stimulus (lower frequency, i.e. 1 to 10 Hz, from Chirp stimulus), and a moving bar (white bar on black background). The alternating full-field stimulus from 1 to 10 Hz was already sufficient to evoke gamma responses up to 60 Hz (Ext. Data Fig. 6), which were also evoked during most other types of visual stimulations, but incapable of producing the increase of power in the high frequency range (Ext. Data Fig. 6J).

## Discussion

In the current experiment, we tested whether it is possible to enhance the power of high-frequency oscillations with spatially organized sequential visual flickering stimulation. We hypothesized that this new stimulation method would allow bypassing the low-pass filter properties of neuronal tissue. In summary, the results from a limited dataset highlight the possibilities for neuronal entrainment beyond full-field flickers. Our results from both single-units and LFPs confirm that lower sequential repeat frequencies entrain stronger responses, hence corroborating the idea that the visual system acts as a low-pass filter. This power loss has been hypothesized to stem from multiple origins, such as synaptic and ion channel noise^18^, active membrane current^19^, neuronal morphology^20^, extracellular space^21^, or even putatively because neuronal capacitance is non-ideal^22^.

Other studies have shown that artificial induction or enhancement of specific brain oscillations can improve cognition. For example, the use of transcranial electric stimulation at 1 Hz during sleep has been shown to enhance memory in healthy humans by increasing slow oscillations and slow spindle activity^23^. 40 Hz full-field flickers improved memory through entrainment of gamma oscillations^4^, although others have challenged such effects^7,8^. Targeting gamma could pose a problem because gamma oscillations are a family of oscillations which have been implicated in many different functions. For example, in mice, broadband and narrowband gamma seem to be two independent oscillations sometimes working in an opposing way^24^: Narrowband gamma is enhanced during running^25^, while broadband gamma is decreased/weakened during running^26 ^what may be the reflect of different regularity of single gamma cycle^27^. Similarly, subtle differences have been observed in humans between sensory evoked and cognition induced gamma, hypothesized to underlie the construction of coherent information representation in possibly top-down manners^28^.

In humans, full-field visual stimulation can entrain steady-state visual evoked potentials (SSVEP) up to approximately 90 Hz^29^. This is likely due to the higher capability of human retinal ganglion cells to respond at such high frequency^30^, compared to mice^8^. Our results intend to give a perspective toward more diverse ways to artificially entrain the brain toward therapeutic effects. This approach is bypassing the listed limits of full-field single-frequency stimulation, hence being able to enhance oscillations at higher frequency. Notably, thanks to our experimental setting, the curved screen covers more than 100° of the visual field of view of the animal. With a given receptive field size ranging from 10 to 25° it permits to sequentially stimulate up to 4–5 neighboring receptive fields independently therefore mimicking the features of neighboring stimulations which could be implemented in corresponding human studies. Humans have receptive fields quite smaller than the ones from mice which are bigger than 15 degrees (Ext. Data Fig. 4), rendering the translation from mice to human vision questionable. In this current study, our stimuli were 10 degrees large (Fig. 1). In human studies, the stimulus design would need to be adapted to the architecture of the human primary visual cortex, for example with smaller stimuli tuned to the smaller receptive field size. This way, the sequential nature of our stimulation could be preserved in both species, as mice stimuli were enlarged to cover several adjacent receptive fields, in order to properly evoke sequential activation.

Interestingly, sequential stimulation with high screen refresh frequency at 180 and 240 Hz failed to increase the high frequency power compared to stimulation with lower screen refresh frequency at 144 Hz (Fig. 3C). This could be due to secondary representations such as orientation tuning and motion coherence, as suggested to occur for gamma oscillations^31^, which should be studied further. The high PPC values for the 144 Hz stimulus indicate that a certain level of neuronal entrainment occurs in V1, which may feed this high frequency power through secondary cortico-cortical connections. We believe that this tendency is due to the longer exposure time permitted by the lower screen refresh frequency, which is also associated with a lower repeat frequency of the complete set of sequential stimuli, especially for the 14 vertical bars compared to the 6 wedges for example (Ext. Data Fig. 5).

Overall, we cannot tell which exact mechanism(s) are producing this enhancement of high frequency power, as it is variable from one stimulus and one animal to the next. We nevertheless observed a significant increase in the amplitude and size from the sequential stimulations compared to checker, chirp and moving bars (Fig. 3E). Surprisingly, these increases of power are homogenic through the cortical mantel rather than initiated by single layer(s) or neuron(s), arguing in favor of a basic phenomenon potentially occurring within cortical sub-layers. Consequently, we propose different alternative hypotheses accounting for this enhancement of high frequency power in mice visual cortex. First, this localized high frequency power increase may correspond to the superposition of the two existing wavefronts between the interconnected primary and secondary visual areas, as the stimulus is sequentially exposed. The receptive field coverage in tangential insertions covers most of the animal retinotopy (> 100°, Fig. 2D). Thus, at the border between primary visual areas, spreading wavefronts may overlap. Second, this enhancement of high frequency power may correspond to an overlap of spreading wavefronts which are known to occur in the visual system of vertebrates upon single point stimulation^32–35^. Consequently, one could hypothesize that the spreading wavefront of the previous sequential stimulation’s section would overlap with the following stimulus section, thereby producing locally higher frequency power in a possibly additive interference. Third, the intrinsic nature of the high frequency sequential stimulus could strengthen the timely co-activation of neighboring neurons, thereby producing the observed higher frequency power enhancement. Importantly, oscillatory stimulation may lead to a higher reliability of the neuronal spiking^36^, therefore higher frequency content can lead to more precise neuronal firing, an effect which is similar to the observed enhanced responses at higher frequency. Nevertheless, the lower pairwise phase consistency for higher frequency speaks against such an interpretation, highlighting the values of randomized frequency as used initially^36^, versus the regular sequential stimuli used in the present study. Last, it is unclear whether feedback interactions between single cells can lead to localized oscillatory activity, which would render them putative underlying actors in the production of the high frequency oscillations we report here.

In the possible outlooks for putative improvement, we list the following points that unfortunately are lacking in the experimental design and the way we acquired them. First, exposing more trials of the stimulation would be a clear way to improve our results. This may be difficult to implement in head-fixed animals which cannot be placed in the set-up for too long. In addition, repeating experiments over several days with repeated acute insertion may not be performed with insertions in the exact same location. Chronically implanted probes would then be a solution, which is feasible for vertical, but likely less for flat tangential insertions which are the ones that best capture the observed enhancement of high frequency power (Fig. 3D). Second, full-field stimuli for the entire range of screen refresh frequencies (144, 180, 244 Hz) and complete set of sequential stimulation repeat frequencies (10, 12, 17, 20, 24, 25, 30, 34, 40 Hz) would be an ideal way to address whether our stimuli could entrain high frequency oscillations in the visual cortex independently, which may be unfruitful as the visual system acts as a low-pass filter^8^, as we observed it ourselves (Fig. 2 & Ext. Data Fig. 3). Such an approach would mimic the fair control of exposing a sampled sinusoidal stimulation at various frequencies. Third, exposing partial sequential stimuli, as of a single section of our stimulus repeatedly, would be a good way to account for putative second-hand SSVEP without the characteristic spatial arrangement present in our stimuli, which will be the subject of our next studies. Furthermore, it would be highly interesting to combine cortical recording with thalamic and collicular recordings to monitor in these lower brain areas – closer to the retina – whether these high frequency oscillations are present. Equally, it is currently unclear whether such high frequency activity would propagate to downstream area(s) such as the retrosplenial cortex, and the hippocampus. Previous reports speak against such a possibility as measured in the gamma frequency^8^. But this does not rule out the possibility that high frequency oscillations in the visual cortex may have a particular influence on downstream regions, particularly the hippocampus.

Finally, we propose extending such sequential approaches to other sensory modalities. Gamma entrainment has already been reported to have different putative therapeutic effects with tactile^37^ and visuo-audio pairing^38,39^. There is a report of pure auditory gamma entrainment using audio stimulation, by multiplexing a 5 kHz carrier into a 40 Hz intensity modulation^40^. Therefore, exploiting the full range retinotopy, tonotopy and somatotopy of sensory stimulation by sequentially activating neighboring receptive fields could become a novel approach to exploit a broader diversity of non-invasive sensory stimulation approaches.

## Materials and methods

### Experimental preparation, animals, Neuropixels probe insertion

All experiments were carried out in accordance with relevant guidelines and regulations, following the approval from the local authorities on the license G 0142/18 (from the German ethics committee: Landesamt für Gesundheit und Soziales Berlin). Great care was taken to minimize the number of animals used, which were all recorded in awake head-fixed condition. The local breeding facility (Charité-Forschungseinrichtung für Experimentelle Medizin) provided the 6 adult C57BL/6J male mice, aged 3 to 6 months from different litters; all part of a single control group. A head-post was implanted 10 days prior to recording, under anesthesia (isoflurane, 2.5% in oxygen Cp-Pharma G227L19A), and complemented with painkillers (metamizole in drinking water, renewed daily) prior to and after implantation (-1 + 3 days). The head-post was fixed with dental cement, which was also used to form a bigger circular crown around the planned insertion to retain phosphate buffer saline solution during recording. The Neuropixels external ground was bound to an Ag/Cl wire fixed directly within this crown to be used as grounding. Habituation was done with daily sessions on the set-up from 30 s to 2 h gradually increased over 5–7 days. Mice were exposed to sparse noise and moving bars a few days prior to the recording day for them to habituate and peacefully stay in head-fixed condition.

On the day of recording, isoflurane was first applied to induce light anesthesia and perform a small craniotomy, either above V1 for vertical insertions, or above the midline areas for tangential insertions, meanwhile adding a ground cable in the crown. The animal could then recover for at least two hours before being placed on the set-up. Stereotactic coordinates were measured from lambda, in the antero-posterior (AP), the medio-lateral (ML), and the dorso-ventral (DV) axes. Vertical insertions were done with a 30°/30° angle from vertical and ML planes, placed in the brain 0 to 0.9 mm AP, + 3 to + 4 mm ML (no DV as pseudo vertical). Tangential insertions were done perpendicular to the midline plane with 15–20° from the azimuthal plane; they enter from the left brain side directly above the retrosplenial cortex: 0 to 0.9 mm AP, -0.5 to + 0.5 mm ML and 0 to -0.2 mm DV from lambda (cf. Figure 1A) as recently published^15^. We detail the corresponding location of the different channels along the probe (Fig. 1B & Ext. Data Fig. 2) along with the location of the neurons that were located within the visual cortices. For both insertions, the probe was placed 4 mm within the brain and then withdrawn 50 μm to release mechanical pressure. The probe was left to settle for ~ 10–20 min before visual stimuli were presented.

Once the recording was finished, the probe positioning was confirmed with DiI (Abcam-ab145311) applied on the probe upon withdrawal and reinsertion after the end of the recording. Once the probe track was stained, the mice were then sacrificed with an excess of isoflurane (> 4%). Cardiac perfusion started with phosphate buffer saline solution and was followed with 4% paraformaldehyde and the brain was sliced and mounted on the next days. 3D reconstruction was done using SHARP-track to relate the probe’s positioning to the Allen Mouse Brain Common Coordinate Framework (Fig. 1B & Ext. Data Fig. 2).

### Hardware, software

Neuropixels probe (version 1.0) signals were recorded with a PXI system (National Instrument NI-PXIe-1071) using Open Ephys software (www.open-ephys.org*).* Consequently, the recordings are systematically composed of 384 channels recorded synchronously at 30 kHz. Furthermore, the recording channels are organized in a checkerboard pattern, with a distance of 20 μm between the 192 recorded rows of channels, and 4 neighboring columns at a distance of 16 µm^12^. Ecephys was used to calculate isolation distance and refractory period violations (Id > 10, Refractory period violation < 0.05% https://github.com/AllenInstitute/ecephys_spike_sorting*).* The rest of the data analysis was performed in Python 3 (www.anaconda.com), using either Wilcoxon signed-rank test with Bonferroni correction when looking into single recording data; where the different neurons are considered as independent (Ext. Data Fig. 3). Or, for the rest of the statistics quantifying the effects in the population of mice, two-way repeated measures ANOVA, followed by pairwise comparison with Bonferroni correction, assuming independence between mice, therefore pooling all neurons within each mouse (Fig. 2, Ext. Data Figs. 3 and 5). “***” indicates a p-value below 0.001, “**”a p-value below 0.01, and “*” a p-value below 0.05. For most analysis all 6 used animals were sampled together unless otherwise stated.

### Sequential and standard visual stimulation

The visual stimuli were displayed on a 27-inch curved gaming monitor screen (Samsung Odyssey G7 27), at different screen refresh rates: 120 Hz for standard stimulation and 144/180/240 Hz for spatially organized sequential stimulations. The screen was gamma corrected beforehand (mean luminance = 120 cd/m²) and placed close to the animal head so that it covered roughly a ±80° area in the azimuthal plane. As previously published in the lab, such positioning of mice and screen can cover sufficient area of the visual field of the mice which revealed itself in the superior colliculus^14^ and in the visual cortex^15^. Such large retinotopic monitoring, which can be bigger than 100° will consequently cover 3–5 non-overlapping receptive fields (Ext. Data. Figure 4). To do so the screen is placed at a distance of 15 cm away from the animal’s eyes, so that 2.6 cm on the screen is equivalent to 10° visual angles from the point of view of the animal eye, on the center of the screen. The curvature of the screen is not perfectly compensating for the warping of the image on the sides of the screen; therefore, it is critical to align the visual stimulus to the center of the field of view of the studied eye, which was done with online analysis of pseudo random presentation of sparse noise as previously published in the laboratory^13^. We did not perform image warping on the screen, leaving some distortion of the stimuli exposed on the most lateral portions of the screen. We exposed all visual stimuli to each mouse, leading to approximately two hours of passive viewing. The standard stimulation list contained a sparse noise stimuli made of either a dark or white square on an opposite background, used to map receptive fields (15° size, 2 targets/frames of 100 ms, Figs. 1B and 20 repeats for each position on a grid of 36 × 22 squares), moving bars (white bars on black background, 10° in width, 12 directions, fixed speed of 90°/s), alternating full-field checkerboards (10° black white full field alternating patterns), and all detailed sequential stimuli. Out of these different stimuli we comparatively reported the evoked spectrum of the white moving bars on black background (fig., 3D), the alternating checkerboards (Fig. 3D), and the alternating full-field stimulus contained in the chirp stimulus (Fig. 3D, detailed in Ext. Data Fig. 6B). The full-field stimulus was produced as follows: starting from a gray background, several light decrement and increment steps were first exposed (2.2 s black, 3.3 s white, 3.3 s black, 2.2 s gray) followed by sinusoidal intensity modulations with increasing frequency (0.5 Hz to 11 Hz) at full contrast (8.75 s) and increasing contrast (0 to 100%) at 0.4 Hz (8.75 s) and ending with 2.2 s gray background. We compared the evoked spectrum to the different sequential stimuli (Fig. 3D). For spatially organized sequential stimulations, 4 different stimulations were presented: top wedges, bottom wedges, vertical bars, and horizontal bars (Fig. 2A). For all sequential stimulations the screen was split into 6, 7, or 14 different sections. The stimulation consisted of alternating black and white checkerboard sections, which were presented sequentially across all neighboring sections. Thus, one section was presented during a single screen refresh cycle, and the next section was presented during the next cycle to produce a seemingly sequential element over the entire screen at different speeds as the screen refresh frequency was changed from 144 to 240 Hz (Fig. 2A). Once all sections are sequentially exposed, the stimulus was repeated for a duration of 2 s for 50 trials alternated with 2 s gray screen. Consequently, the repeat frequencies of the complete set of sequential stimuli were 10.3/12.9/17.1 Hz for the 14 vertical bars, 20.6/25.7/34.3 Hz for the 7 vertical bars and 24/30/40 Hz for both wedge types, at 144/180/240 Hz respectively (Ext. Data Fig. 5 for details). For all animals, all stimuli were systematically exposed within a single recording session within the same data on the same screen. The sequential stimuli were exposed either before or after the standard stimuli which were each randomized.

### LFP analysis

The LFP analysis was performed on the 2.5 kHz signal, starting by applying a band pass Butterworth order 3 filter (Scipy, > 0.1 Hz, < 400 Hz), then down sampled at 1250 Hz by averaging neighboring data points. Evoked LFPs were produced by averaging all repeats of the same visual stimulus (Ext. Data Fig. 6), to further study the 2 s long SSVEP responses observed in the brain. Evoked PSDs were illustrated using the Welch function (Fig. 3A, scipy). Most of the analysis done on the temporary evoked spectrum was done using another function because of the limits of resolution that the Welch function offers: the evoked spectrum was extracted on a channel-to-channel basis using a Stockwell function to observe over time the changes of power (https://github.com/claudiodsf/stockwell, Fig. 3B & Ext. Data 6). This method, which stems from geophysics^40^, is a generalization of the short-time fast Fourier transform (here implicitly defined in a given channel ‘ch’ as the derived phase correction of the continuous wavelet transform with window being the gaussian function^40^):

$$\:S_{x} \left( {t,f} \right) = \int \: _{{ - \infty \:}}^{{ + \infty \:}} x\left( {\tau \:} \right)\left| f \right|e^{{ - \pi \:(t - \tau \:)^{2} f^{2} }} e^{{ - j2\pi \:f\tau \:}} d\tau \:$$

A Z-score of evoked activity was then calculated within each channel between baseline and the SSVEPs period (black thick line Fig. 3B, bottom of each plot from 0 to 2000 ms) in relation to its pre-stimulus baseline (-500 ms to -5 ms, Fig. 3), and then further quantified as a Z-score evoked spectrum (Fig. 3C).

$$\:{Z}_{score\:\left(stim\:a\right)}\left(f,ch\right)=\frac{{S}_{stim\:a}\left(t=\:\left[0:2000\right],\:f,\:ch\right)-\:mean\left({S}_{stim\:a}\left(t=\:\left[-500:-5\right],\:f,\:ch\right)\right)}{{S}_{stim\:a}\left(t=\:\left[0:2000\right],\:f,\:ch\right)+\:mean\left({S}_{stim\:a}\left(t=\:\left[-500:-5\right],\:f,\:ch\right)\right)}$$

We then used this 2D Z-score (Fig. 3C) to further quantify the baseline-to-signal increase of power for each frequency and each channel. The high frequency band of 100–190 Hz was chosen to quantify the strength of evoked high frequency events (Fig. 3C, D, E). The profile of the Z-score evoked spectrum was then estimated (Fig. 3C) to determine the size and strength of enhanced high frequency power. To quantify the amplitude and size of the enhanced high frequency power, the absolute values of the cumulative 2D z-score were reported for each stimulus, including the standard visual stimulations (Ext. Data Fig. 6): checkerboard mapping, chirp, and the 12 different orientations of the moving bar stimulus (Fig. 3C-E, Ext. Data Fig. 6).

$$\:Amp\:\left({Z}_{score\:\left(stim\:a\right)}\right)=Area\:under\:the\:curve\:\left(abs\left(\sum\:_{ch=284\:or\:50}^{ch=384\:or\:250}{Z}_{score\:\left(stim\:a\right)}\left(f,ch\right)\right)\right))$$

$$\:Size\:\left({Z}_{score\:\left(stim\:a\right)}\right)=Number\:of\:channels\:above\:15\:a.u.\:\:\left(abs\left(\sum\:_{f=90}^{f=180}{Z}_{score\:\left(stim\:a\right)}\left(f,ch\right)\right)\right)$$

### *Multi-unit and* Single-unit extraction and analysis

To extract the multi-unit activity (MUA), we first applied a median subtraction on the raw action potential band signals recorded from the Neuropixels probe (across channels and time) followed by a bandpass filter (Butterworth filter order 2, 0.3 to 3 kHz). Consequently, multi-unit extraction was realized by selecting any signal passing the threshold of 4 standard deviations above that of the given channel.

Single-unit sorting was done with Kilosort 2.5 (https://github.com/MouseLand/Kilosort) using MATLAB 2018 (www.mathworks.com*).* Manual inspection merging and curing clustering was done in Phy2 (https://phy.readthedocs.io/en/latest/*).* Clusters of high quality were kept after applying quality metrics thresholds (Isolation distance > 10, ISI_violation < 0.05%). Broad waveform (putative excitatory) neurons were distinguished from narrow waveforms (putative inhibitory) neurons based on the shape of the waveforms and their peak-to-trough duration (0.43 ms, Ext. Data Fig. 1). This separation of waveform is a crude approach to distinguish between excitatory and inhibitory neurons, which possibly cover a distinction between Pval, and Sst neurons versus all others when using optogenetics^42^, which is still being questioned when using ultra-high density silicone probe^43^. For the quantification of the visually driven firing responses of both BW and NW neurons, we first added a threshold on the responsiveness of the neurons. To do so, we produced a peri-stimulus time histogram of overlapping signal from the different trials when aligned to the onset of the different stimuli. Next we defined the baseline as the pre-stimulus baseline (-500 ms to -5 ms, Fig. 3) to then observe whether the evoked activity that we measured (500 ms to 2 s, Fig. 3) reached 7.5*SNR of the baseline (Ext. Data Fig. 3). In two of our six recordings, multiple behavioral noise artifacts (possibly stemming from grounding contact within the crown) were present in the recordings (outside of the analyzed visual stimulations) which strongly impaired the quality of the obtained single-units, and hence they decrease firing rate over time (Ext. Data Fig. 3). However, these recordings were kept for the pairwise correlations and LFP analysis.

Both for single-units and for MUA, receptive fields were estimated using spike-triggered averages (STA). A 2D-cubic-interpolation (scipy. interpolate.interp2d) was used to approximate the exact receptive field shape and their size was then approximate as their width at half their individual peak amplitude. Their center was estimated from the peak of their receptive field. Their cumulative azimuthal and elevation extent was calculated by the Euclidean distance between the receptive field center.

Pairwise phase consistency (PPC) was estimated by measuring the time/phase difference from a fixed sinusoid signal aligned to the start of the stimulus at the given stimulus repeat frequency (Ext. Data Fig. 5). For each neuron’s spikes, the theta values were estimated as the phase position of the artificial oscillation for the stimulus (Ext. Data Fig. 5A). To estimate a single spike phase position, we calculate the time position of the spike in the fixed sinusoid which we divided by the period duration making it a unit-less quantity.

$$\:For\:Neuron\:n,\:given\:3\:spikes,\:we\:estimate\:{\theta\:}_{\mathrm{1,1}}\:\&\:{\theta\:}_{\mathrm{1,2}\:}\&\:{\theta\:}_{\mathrm{1,3}}$$

Pairwise values were then evaluated as the combination of all pairwise values between the spikes and the oscillation within a recording, the sum of which was then calculated for each neuron and then averaged within each recording (Fig. 1D & Ext. Data Fig. 5B), and dataset (Ext. Data Fig. 5C)^8^.

$$\:PPC\:\left(N\:neurons\right)=\:\frac{2}{N\left(N-1\right)}\mathrm{*}\sum\:_{j=1}^{N-1}\sum\:_{k=j+1}^{N}\mathrm{cos}\left({\theta\:}_{j,k}\right)\mathrm{*}\mathrm{cos}\left({\theta\:}_{j,k}\right)+\mathrm{sin}\left({\theta\:}_{j,k}\right)\mathrm{*}\mathrm{s}\mathrm{i}\mathrm{n}\left({\theta\:}_{j,k}\right)$$

## Electronic Supplementary Material

Below is the link to the electronic supplementary material.

Supplementary Material 1

## Data Availability

Data and scripts will be provided freely upon reasonable request.
