# Supplementary material for: Sequential visual stimuli increase high frequency power in the visual cortex
Source: Sci Rep. 2026 May 17;16:15228. doi: 10.1038/s41598-026-52253-9 (PMC13181131; doi:10.1038/s41598-026-52253-9)
Supplement: Supplementary file 1 — Supplementary Material 1 [file 41598_2026_52253_MOESM1_ESM.docx]

**SUPPLEMENTARY MATERIAL**


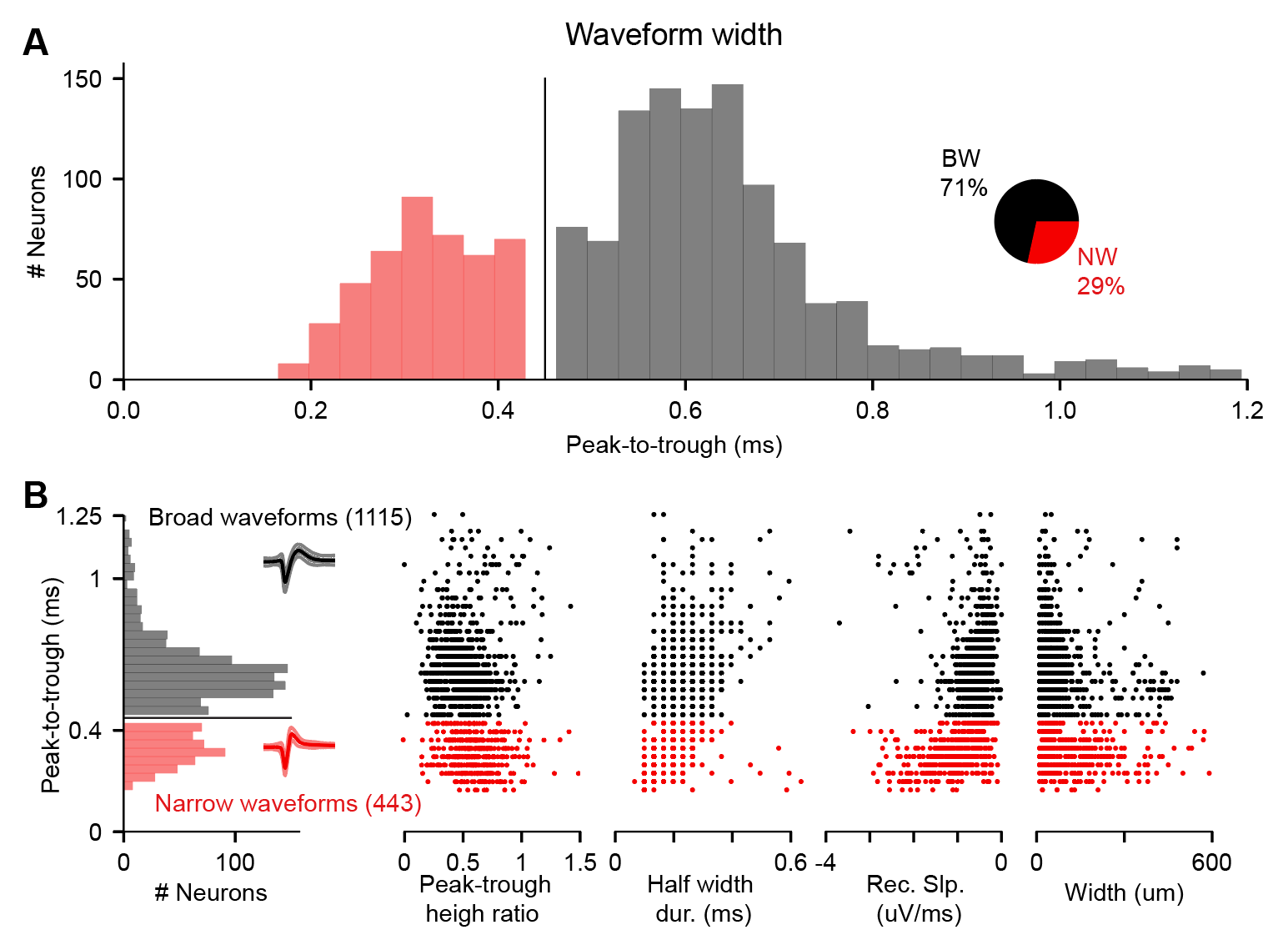


**Extended Data Figure 1: Separation of single-unit waveforms in broad waveforms (BW, putative excitatory) versus narrow waveforms (NW, putative inhibitory) neurons.** **A**. Distribution of the waveform duration of the 1558 single units obtained from all recorded regions (n = 6 mice, visual cortex and deeper structures). **B.** Simple quantification of single-unit properties: peak-to-trough ratio, half-width duration, recovery slope and spatial width.


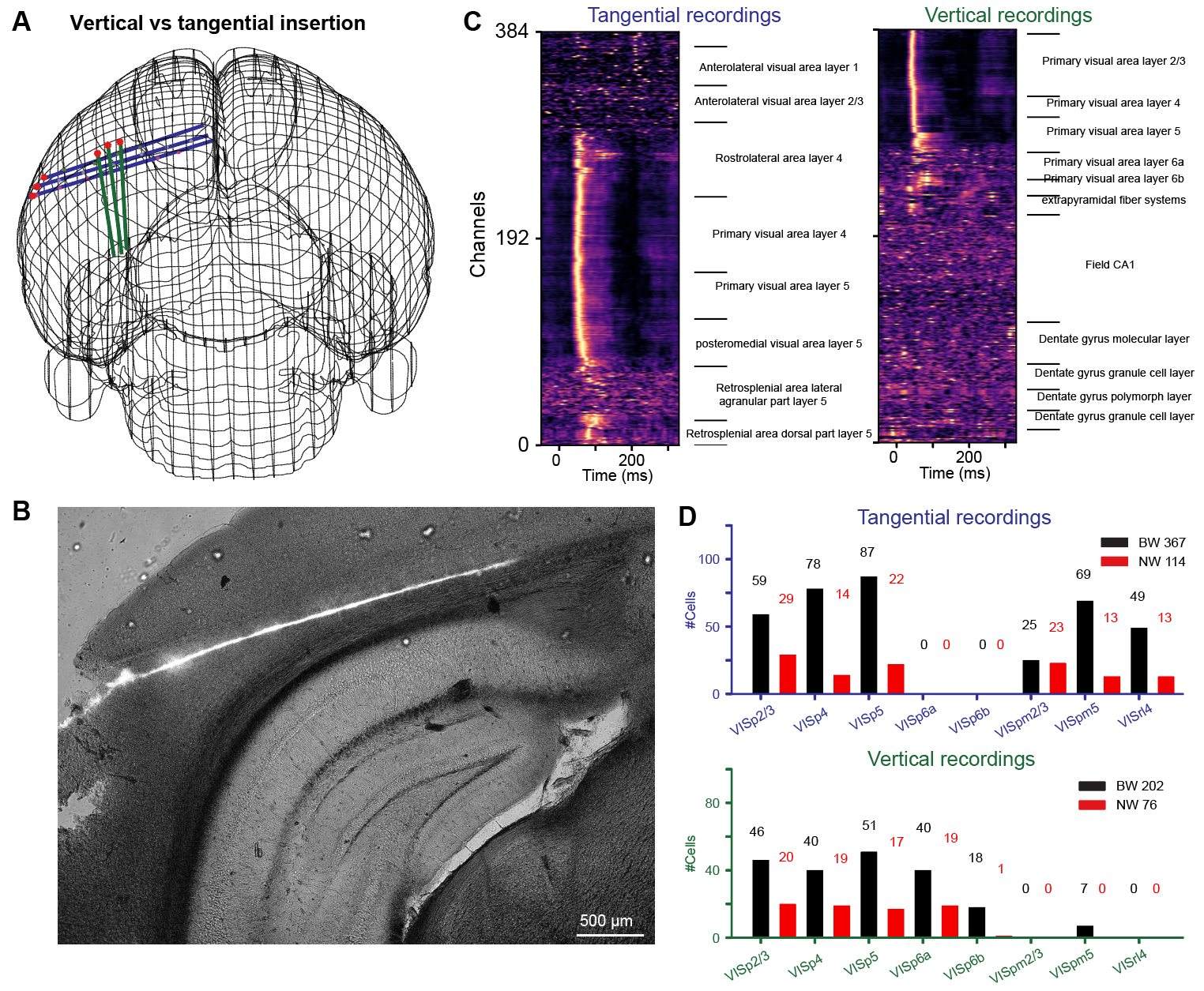
  **Extended Data Figure 2: Illustration of the recording placement and corresponding single neuron locations.** **A**. Sharp-track reconstruction of 3D mice brain to illustrate our 6 recordings. **B.** Histological picture of a tangential Neuropixels insertion, with pseudo color corresponding to the DiI staining. **C.** Aligned multi-unit activity of a tangential insertion through the visual layer of the cortex (left) and a vertical insertion through the brain (right), with the corresponding layer to layer location (right). **D.** Quantification of the different BW and NW neurons in the visual layers only, for tangential insertions (top) and vertical insertions (bottom).


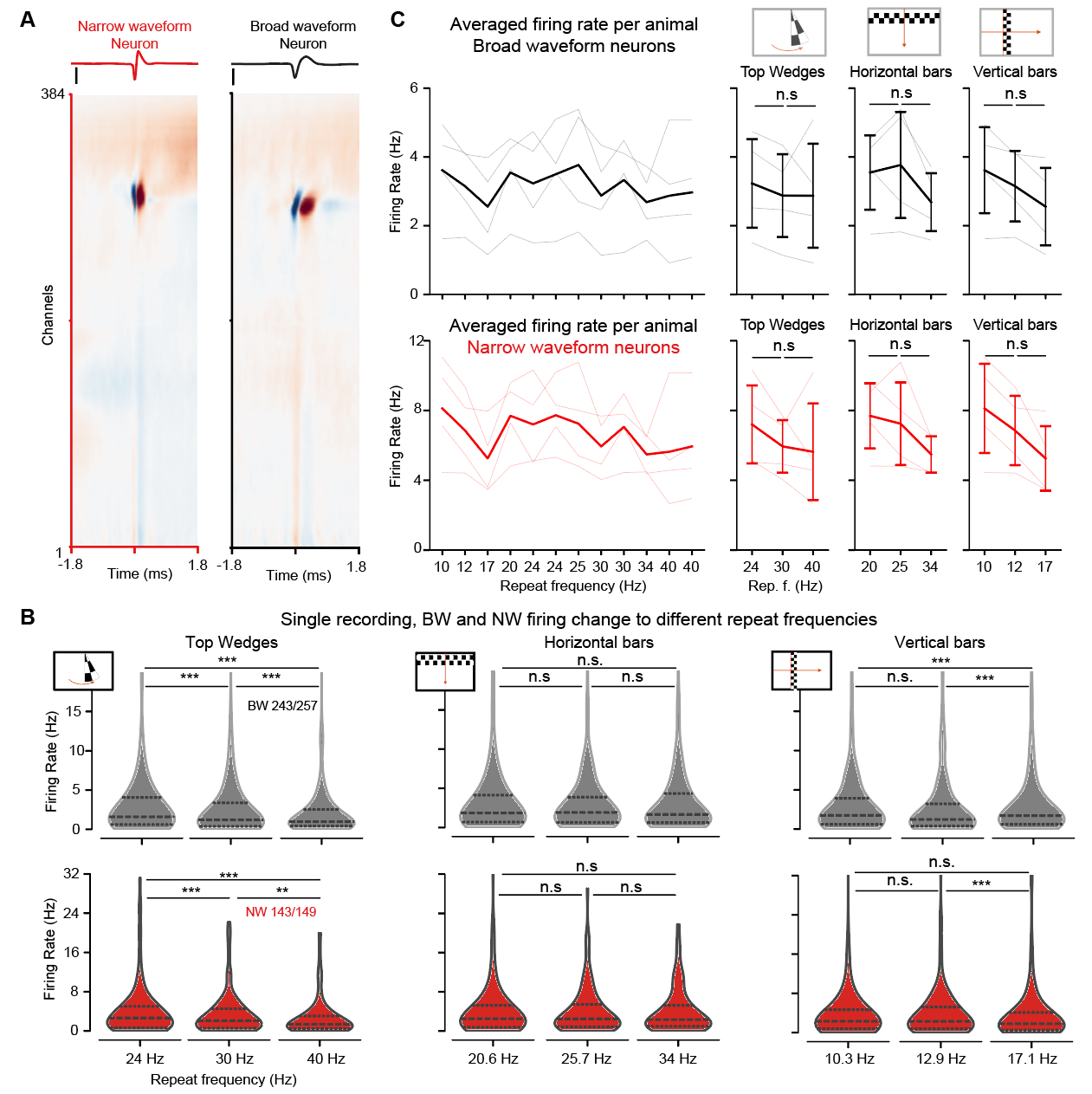


**Extended Data Figure 3: Higher frequency repeats in the sequential stimuli are associated with a reduction of firing of both BW and NW neurons.** **A.** Multichannel waveforms of a NW and a BW neuron. **B.** Firing rate changes in a single recording for increasing repeat frequencies in response to top wedges (left), horizontal bars (middle), and vertical bars (right). In both BW neurons (top black), and NW neurons (bottom red), the evoked activity was smaller for higher frequency repeats. Two-sided Wilcoxon signed-rank test (*** < 0.001, * < 0.05), with Bonferroni correction. **C.** Same results in 4 different animals (thin lines) with their respective inter-animal averages (thick lines). (n = 371 NW, n = 951 BW, pooled within each of the n = 4 mice, Two ways ANOVA). Graphs show median, interquartile range (IQR), and range with Seaborn plots, otherwise mean and SEM.


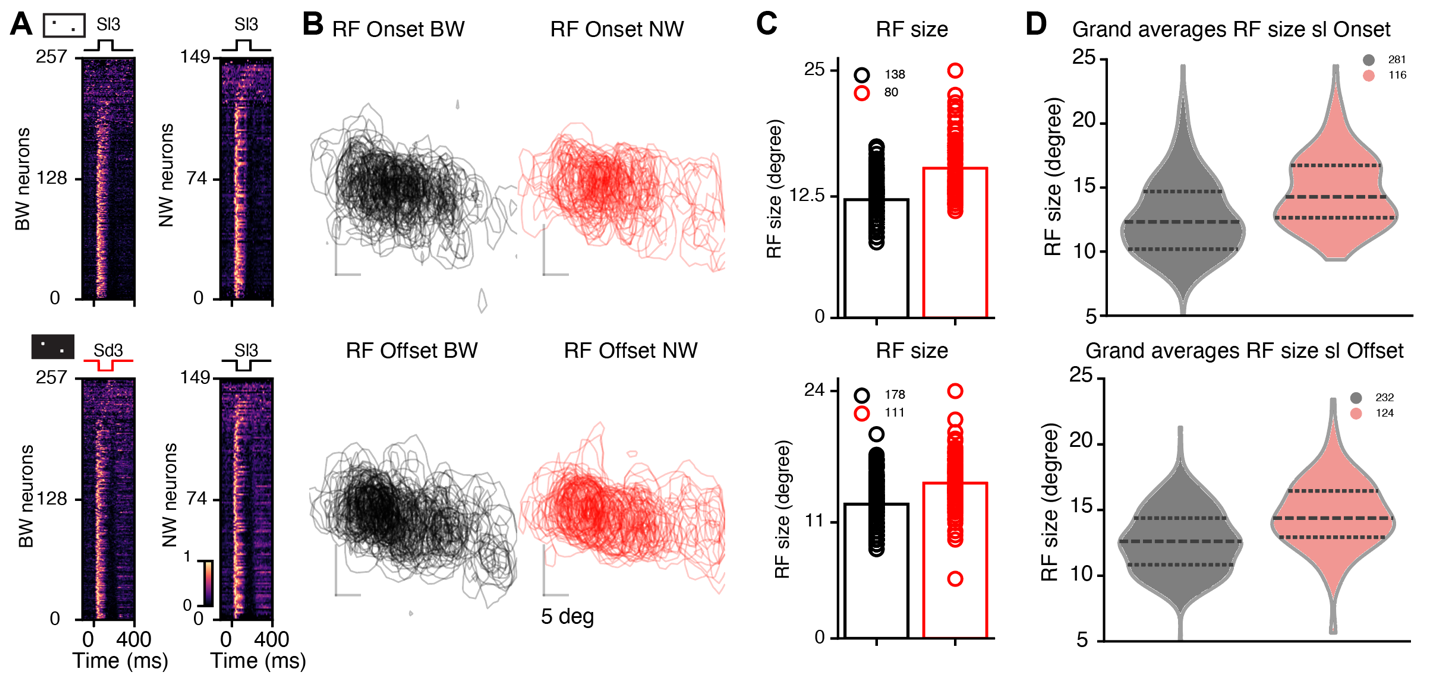


**Extended Data Figure 4: Onset and Offset receptive field quantifications.** **A.** Visually driven activity in BW waveforms (left columns) versus NW (right~~er~~ columns) of all neurons within one recording for onset responses (light on dark, top row) and offset responses (bottom row). **B.** Above threshold receptive field obtained in a tangential recording for BW (left columns) and NW (right column) for onset responses (top row) and offset responses (bottom row). **C.** Half peak size quantification of the same receptive fields for onset responses (top) and offset responses (bottom) in one recording. (n = 80/149 NW, n = 136/257 BW, for onset RF, n = 111/149 NW, n = 178/257 BW for offset responses). **D.** Quantification of all receptive field sizes obtained in the dataset for all neurons that pass thresholds within visual areas (n = 116/190 NW, n = 281/569 BW, for onset RF, n = 124/190 NW, n = 232/569 BW for offset responses, n = 6 mice).

**
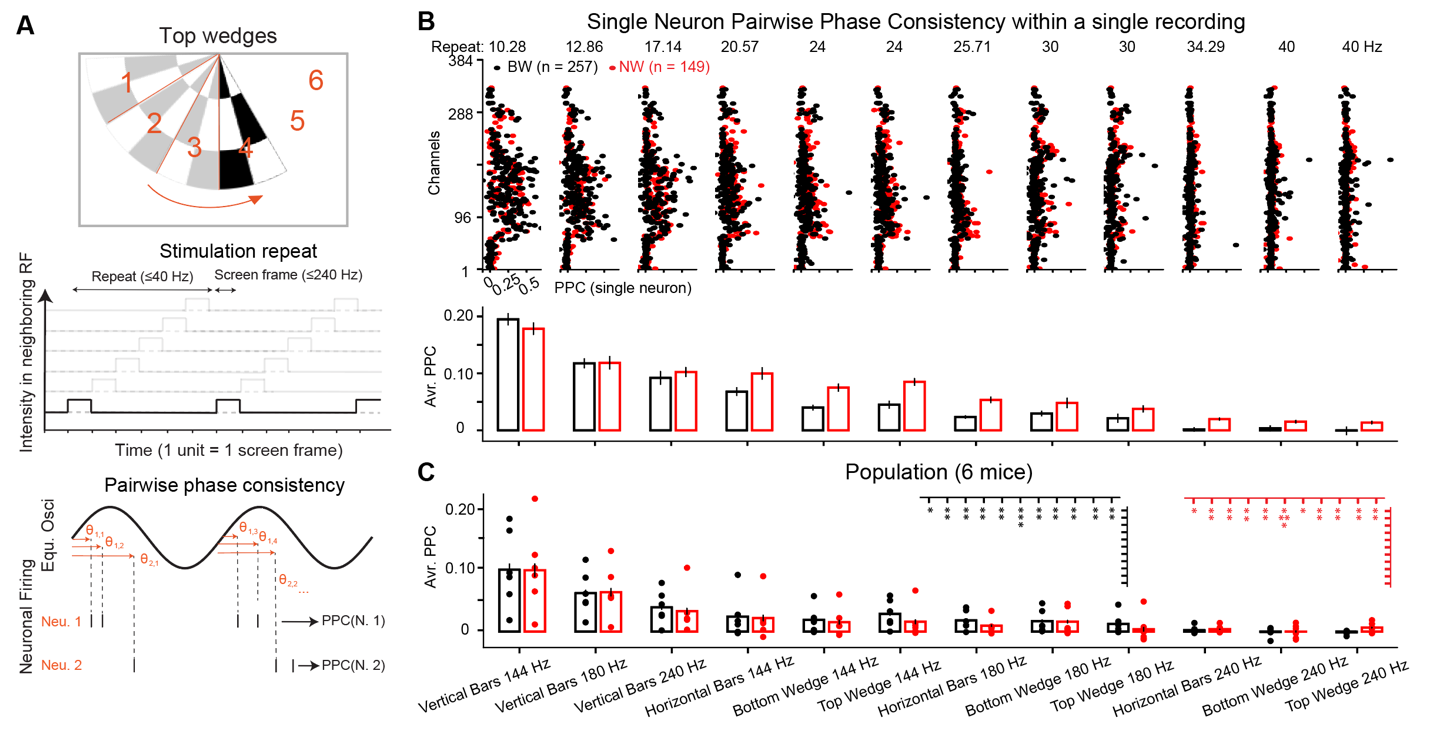
**

**Extended Data Figure 5: Pairwise phase consistency based on each stimulus repeat frequency.** **A.** Schematic of the pairwise phase consistency extraction, one example stimulus (top), the sequential motif resulting from the stimulation (middle), and the equivalent theoretical oscillation upon which the phase of each individual neuron’s spikes is evaluated (bottom). **B.** Channel position of BW and NW PPC (top) and their average population condition (bottom). **C.** Dataset averages of the PPC of BW and NW neurons in V1 along the different repeat frequency content of the different sequential motif combinations, two way repeated measures ANOVA, p = 7.3 10^-3^, for BW, p = 8.5 10^-12^, for NW. Post hoc pairwise comparison with Bonferroni correction reports a significant difference (p < 0.05, n = 1115 BW, n = 443 NW, pooled within each of the n = 6 mice) within the vertical bars and toward all others. Note that the screen frequency refresh is reported in the lower x-axis.


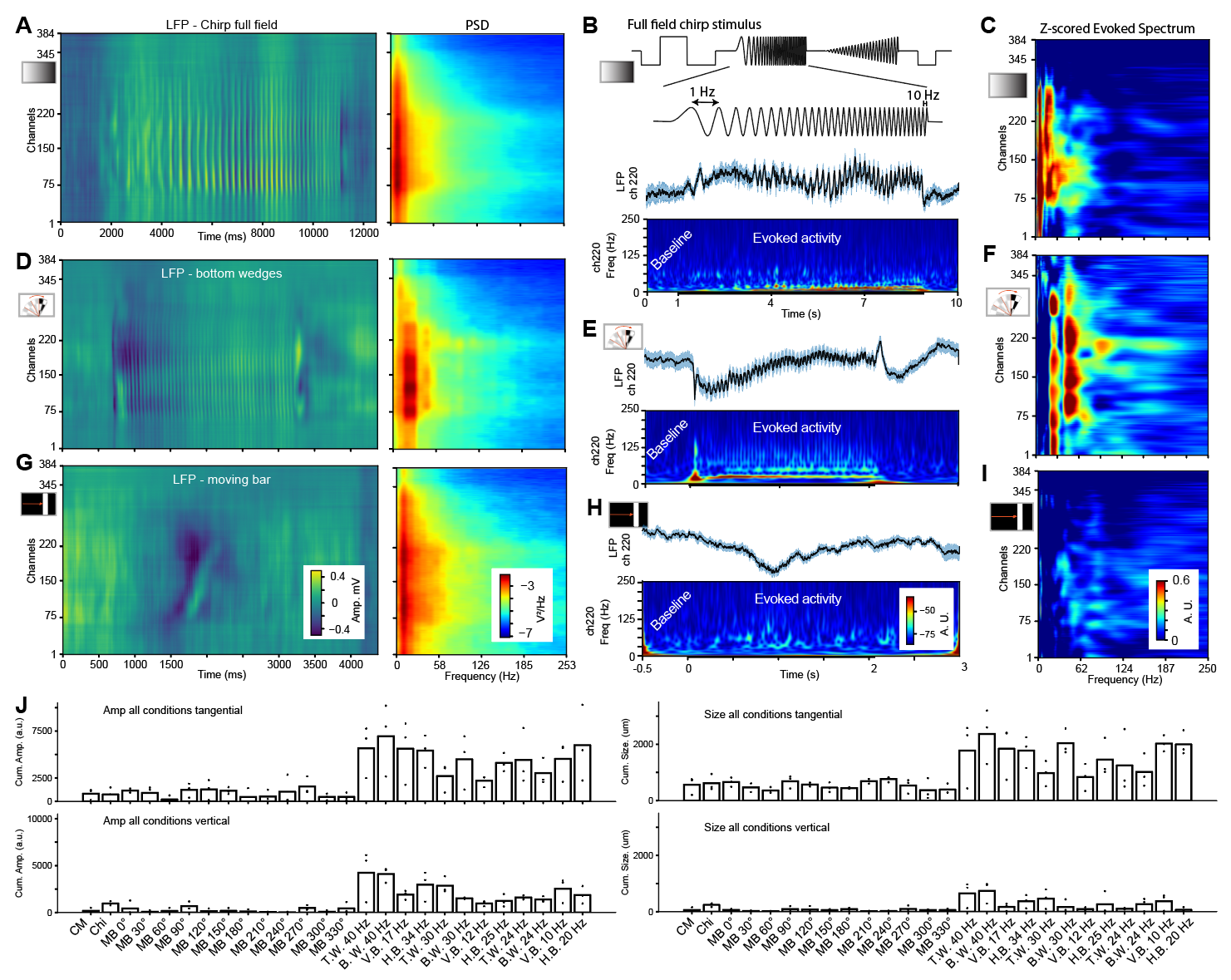


**Extended Data Figure 6: Comparing evoked frequency content of flickering full-field from chirp stimulus and moving bar versus sequential stimulation.** **A-C**. Evoked responses from Chirp stimulations. **A.** LFP during the frequency increase of the chirp stimulus (left), PSD of the evoked LFP during a full-field flicker (right). **B.** Full-field chirp stimulus (top) is made of a combination of frequencies and intensity changes, which contain a regular 1 to 10 Hz full-field flicker period (middle top trace), with the corresponding LFP in the most responsive channel (220, lower top trace, inter trial SEM is indicated shaded blue) and the corresponding spectrogram (lower panel). **C.** Corresponding full-field evoked Z-score (right). **D-F.** Similar plotting as in A-C for sequential bottom wedge stimulus. **D.** LFP, and PSD in response to the bottom wedge’s stimulus, plotted on similar times as below. **E.** LFP (top) and Spectrogram (bottom). **F.** Related evoked z-score spectrum. **G-I**. Similar plotting as in **A-C** for moving bar stimulus. **G.** LFP, and PSD. **H.** LFP (top) and Spectrogram (bottom). **I.** Related evoked z-score spectrum. Note the little differences of apparent content in the different PSD between stimuli, meanwhile the Z-score evoked spectrum using Stockwell extraction reveals the expected high frequency power increases. **J.** Quantification of the cumulative absolute z-score spectrum increase, in the visually driven areas (Ch. 284 to 384 in vertical, and Ch. 50 to 250 in tangential insertions), of the high frequency windows (90 to 180 Hz). Each condition’s values are reported for each mouse in a corresponding dot, and their averages in bars, for amplitudes (left), sizes (right), for tangential insertion (top row) and vertical insertions (bottom row), for all 12 moving bars orientation and the 12 different sequential stimulations. Each sequential stimulation is distinguished by the screen position repeat frequency.
